# Identification of *Veratrum* Species in Pimacao Based on ITS2 Sequences and Steroidal Alkaloids by a Pseudo-Targeted Metabolomics Method

**DOI:** 10.3389/fpls.2022.831562

**Published:** 2022-04-11

**Authors:** Qinwei Lu, Shuaiyao Wang, Zili Yin, Qinsheng Chen, Xingchao He, Qi Wang, Qingyu Hu, Yu Gu, Huiru Tang, Hui Xie

**Affiliations:** ^1^Zhangjiang Fudan International Innovation Center, Human Phenome Institute, Fudan University, Shanghai, China; ^2^Yunnan Key Laboratory of Dai and Yi Medicines, Yunnan University of Chinese Medicine, Kunming, China; ^3^Yunnan Baiyao Group Co., Ltd., Kunming, China; ^4^University of Chinese Academy of Sciences, Beijing, China

**Keywords:** Pimacao, *Veratrum*, ITS2, steroidal alkaloids, pseudo-targeted metabolomics method

## Abstract

Pimacao is a traditional Chinese folk medicine and is the main component of the famous Chinese herbal remedy “Yunnan Baiyao” for its significant analgesic activity in the treatment of wounds. Due to increases in consumption, its wild population is now difficult to find, and adulterant from the same genus has occurred. However, this is challenging to distinguish the species of *Veratrum* in Pimacao using dried roots and rhizomes or medicinal powder. ITS2 sequences and steroidal alkaloids by the non-targeted and pseudo-targeted metabolomics methods were taken advantage of establishing an effective identification method. Based on the ITS2 sequence, metabolite profiling of steroidal alkaloids and morphological characteristics, the classification of two distinct subspecies in *V. mengzeanum* has been reinforced. In addition, the new subspecies *V. mengzeanum* subsp. *phuwae* was collected in China for the first time. The ITS2 sequence could be used in the identification of *V. taliense*, *V. mengtzeanum*, *V. stenophyllum*, and *V. nigrum*, but is insufficient for intraspecific identification. Simultaneously, 147 variables were labeled by non-targeted analysis accomplished utilizing an ultra-high-performance liquid chromatography electrospray ionization orbitrap tandem mass spectrometry (UPLC-ESI-QE-Orbitrap-MS) system consisting of an Orbitrap QE HF-X. Followed by a pseudo-targeted analysis method developed for the Qtrap 6500-plus mass spectrometry system coupled with an ESI source, 29 labeled steroidal alkaloids detected by the MRM mode could distinguish between four species. Notably, 25 labeled steroidal alkaloids could distinguish between three closely related species. These have the potential to be used as markers for identification. Furthermore, there were several variables with statistical differences between two subspecies of *V. mengtzeanum* and populations of *V. taliense*, *V. mengtzeanum*, and *V. stenophyllum*.

## Introduction

Pimacao is a traditional Chinese folk medicine and is the main component of the famous Chinese herbal remedy “Yunnan Baiyao” for its significant analgesic activity in the treatment of wounds. It has also been used in other Chinese formulated products, such as “Yili Zhitong Dan,” for the alleviation of pain, including cancer pain and acute pain. The dried roots and the rhizomes of a few *Veratrum* species, such as *V. taliense* Loes., *V. stenophyllum* Diels, *V. mengtzeanum* Loes., and *V. grandiflorum* (Maxim.) Loes. f., have been used as Pimacao (Medical Products and Food Administration of Yunnan Province, 2005). These plants are generally found in northern Yunnan province and southwestern Sichuan province in China. However, the understanding of differences in the medicinal activity of these species is limited. Simultaneously, as consumption of these plants increases, their wild population becomes harder to find. Adulterant with the same genus has also occurred due to the similar shape of roots and rhizomes. In particular, this has been observed with *V. nigrum* L., which is the source of traditional Chinese medicine “Lilu.” However, “Lilu” has emetic and hypotensive effects. Therefore, it is necessary to establish an effective identification method to distinguish between the various species of *Veratrum* using dried roots and rhizomes or medicinal powder.

*Veratrum* are perennial herbs belonging to the Melanthiaceae family and are distributed in the temperate zones (occasionally extending to arctic zones) of the northern hemisphere (The Catalogue of Life Partnership, 2017). They were formerly considered to be part of the Liliaceae family (Chen and Hiroshi, 2000). The nuclear ribosomal internal transcribed spacer (ITS) sequence, including the ITS1-5.8S-ITS2 sequence, has been used in the phylogeny and biogeography of *Veratrum* (Liao et al., 2007; Kim et al., 2013). The second internal transcribed spacer (ITS2) ribosomal DNA (rDNA) sequence is a widely used molecular marker for the identification of species due to its observed concerted evolution (Chen et al., 2010; Anaz et al., 2021). Therefore, the establishment of a molecular phylogeny of Pimacao original plants using ITS2 sequences could lead to meaningful conclusions, and provide an important basis for quality control.

Previous phytochemical and pharmacological investigations suggested that the main active compounds of the genus *Veratrum* are steroidal alkaloids (Li et al., 2006; Chandler and McDougal, 2014). In addition to teratogenic toxicity, the anti-inflammatory, analgesic, antioxidant, antihypertensive, antiplatelet, and antitumor activities of *Veratrum* plants have been reported (Li et al., 2016; Dumlu et al., 2019; Zhang M. Z. et al., 2020; Xie et al., 2021). In particular, many have been shown to be antagonists of the Hedgehog (Hh) signaling pathway, a common target for anticancer therapy (Li et al., 2016; Ghirga et al., 2018). Until now, nearly 200 steroidal alkaloids have been reported from different species (Li et al., 2020; Zhang M. Z. et al., 2020; Gao et al., 2021). They are categorized into cevanine (A), veratramine (B), jervanine (C), solanidine (D), and verazine (E) types according to their carbon framework (Liang, 1984), as shown in Supplementary Figure 1.

A non-targeted method using high-resolution mass spectra could simultaneously analyze as many metabolites as possible in the samples without bias. However, this is challenging as a quality control method due to the limited repeatability within instrument and laboratory. In contrast, a targeted method could be used to detect particular compounds with stability and repeatability, but this is limited by the number of known compounds. A pseudo-targeted method integrating the merits of non-targeted profiling and targeted detection selects two or more ion pairs for the detection of one metabolite. Here, the most abundant ion is used for quantitative analysis, while the others are used for qualitative analysis (Li et al., 2012; Zhang J. et al., 2020).

In this study, the ITS2 and the steroidal alkaloids of Pimacao by the pseudo-targeted method have been utilized to establish an effective identification method to distinguish *Veratrum* species in Pimacao, even among the populations. It will provide possible approaches for promoting the quality control level and safety use of Pimacao.

## Materials and Methods

### Plant Materials

**FIGURE 1:**
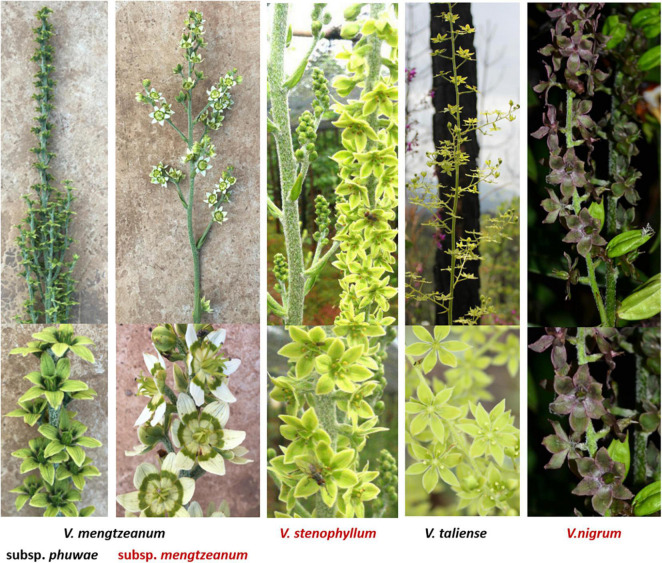
Inflorescence of Pimacao origin plants.

Plant materials were collected from nine different populations in the Yunnan and Guizhou provinces (Table 1). For each population, more than six plants were randomly collected at least 5 m apart. The plants were identified by Dr. Hui Xie, and the voucher specimen from each population was deposited in the herbarium of the museum of the Yunnan University of Chinese Medicine. The specimens were described in Table 1. The inflorescence of taxa was photographed and shown in Figure 1.

**TABLE 1:** Information of samples.

Populations	Species	Voucher	Location	Longitude	Latitude	Altitude/m	GenBank accession numbers
BX	*V. taliense*	BS090701	Longyang, Baoshan, Yunnan	99°17′	25°12′	2,800	MG745765
BY	*V. taliense*	BS090702	Wayao, Baoshan, Yunnan	98°05′	25°03′	3,150	MG745766
XY	*V. taliense*	XP081402	Xingping, Yuxi, Yunnan	102°01′	24°04′	2,150	MG745771
CB	*V. stenophyllum*	DLBL	Cangshan, Dali, Yunnan	100°09′	25°38′	2,720	MG745776
DX	*V. stenophyllum*	1-DLTH	Taihe, Dali, Yunnan	100°24′	25°45′	2,800	MG745774
GJ	*V. mengtzeanum* subsp. mengtzeanum	GJ15082101	Gejiu, Honghe, Yunnan	103°12′	23°22′	2,250	MG745768
MZ	V. mengtzeanum subsp. *mengtzeanum*	MZ090601	Mengzi, Honghe, Yunnan	103°41′	23°36′	2,350	MG745769
XJ	*V. mengtzeanum* subsp. *phuwae*	XP081401	Xingping, Yuxi, Yunnan	101°71′	23°40′	2,750	MG745770
GB	*V. nigrum*	HZ151030	Bijie, Guizhou	104°46′	26°58′	2,170	MG745767

After being cleaned with a brush, the fibrous roots and rhizomes were removed from the plants and immediately dried with allochronic silica gel in a self-contained bag. Once the mass stabilized, the samples were cut into pieces with scissors and ground into powder using an SKSI tissue lyser (BiHeng Biotechnology Inc.) at 60 Hz for 30 s for a total of three cycles. Ground samples were passed through a 40-mesh sieve and stored in well-sealed sample bottles at 4°C until further analysis.

### Chemicals

Acetonitrile (ACN) and formic acid (FA) were purchased from Sigma-Aldrich (MO, United States) and methanol (MeOH) was obtained from Merck & Co. Pure water was prepared using a Milli-Q system (Millipore, MA, United States). All solvents and reagents were of analytical grades.

### Molecular Phylogenetic Analysis Based on Second Internal Transcribed Spacer Sequence

Plant genomic DNA was extracted from 20 mg of plant powder (six plants/population) utilizing the AxyPrep™ Multisource Genomic DNA Miniprep Kit 50-prep (Axygen, United States). DNA mass and concentration were measured using a nucleic acid and protein analyzer (BioPhotometer plus, Eppendorf, Germany).

Primers used for PCR included “ITS2 F” (5′-ATG CGA TAC TTG GTG TGA AT-3′) and “ITS R” (5′-GAC GCT TCT CCA GAC TAC AAT-3′), as reported previously (Chen et al., 2010) and synthesized by Invitrogen™ (Thermo Fisher Scientific, China). PCR samples were made by mixing 2.0 μL 10 × PCR buffer, 1.0 μL dNTP (2.5 mmol/L each), 0.25 units of Taq polymerase, 2.0 μL primers (10 μmol/L) each, 1∼4 μL template, and ddH_2_O to a final volume of 20 μL. PCR amplification was conducted by first heating the samples to 95°C for 5 min before repeating 30 cycles of: 95°C for 45 s, 60°C 45 s, and 72°C 45 s. Samples were then held at 72°C for 10 min. After purification, the PCR products were sequenced directly with an ABI 3730 sequencer (Invitrogen™, Thermo Fisher Scientific).

The acquired sequences for each sample were compared using the ClustalX version 1.83 to predict a strictly aligned region as the representative sequence. MEGA6 (Tamura et al., 2013) was then used to determine the sequence homology between each sample. Reliability was tested by bootstrap with 1,000 repeats. Evolutionary divergence between sequences and species was presented using MEGA6 also (Yang et al., 2020).

### Non-targeted Metabolomics Analysis

Plant powder (six plants/population) was individually freeze-drying under vacuum and 50 mg of powder from each plant were precisely weighed. After 1 mL methanol solution containing 0.15% formic acid (FA) was added, samples were vortexed for two min. Then, the samples were placed on ice for 30 min before they were subjected to ultrasound (80 Hz, 5 min) in an ice bath. After that, each sample was placed in a metal bath (30°C, 900 rpm) for 30 min, followed by centrifugation at 12,000 rpm for 5 min. The supernatant was collected and filtered through a 0.22 μm PTFE membrane filter for subsequent analysis. Ten microliters from each sample was mixed to generate a quality control (QC) pooled sample. During analysis, one QC sample was injected after every six samples running, to ensure the data stability.

Non-targeted analysis was accomplished with the ultra-high-performance liquid chromatography electrospray ionization orbitrap tandem mass spectrometry (UPLC-ESI-QE-Orbitrap-MS) system consisting of an Orbitrap QE HF-X (Thermo Fisher Scientific, Germany) and a Vanquish UPLC (Thermo Fisher Scientific, Germany). Separation was performed on a ZORBAX RRHD Eclipse Plus C18 column (100 mm × 2.1 mm, 1.8μm) at 40°C with the mobile phase (A) 0.15% FA in water and (B) 0.15% FA in acetonitrile/methanol (1:1, v/v). The gradient started from 10 to 20% B for 2 min, gradually increased from 20 to 45% B at 2–25 min, followed by a steeper 5 min linear gradient from 45 to 100% B before returning to 10% B by 30–30.1 min and fixed for 4.9 min for column equilibration. The flow rate was set at 0.35 mL/min. Mass spectra were recorded using an electrospray ionization (ESI) source in positive ionization mode. Full scan mode was obtained from m/z 150 to 2,000 with a resolution of 60,000, while data-dependent MS/MS (dd-MS2) mode was acquired at a resolution of 15,000, with collision energy at 25 eV. The instrument parameters were set as follows: Spray voltages were 3500 V and the capillary and probe heater temperatures were set to 320 and 350°C, respectively. Sheath gas and aux gas flow rate were 50 and 10 (in arbitrary units), respectively, and the S-lens RF level was 60 (Roiffé et al., 2019).

### Pseudo-Targeted Metabolomics Analysis

Samples for pseudo-targeted analysis (Du et al., 2017; Xuan et al., 2018) were prepared identically to the non-targeted approach. The pseudo-targeted method was conducted using the Qtrap 6500-plus mass spectrometry system (AB Sciex Corp., United States) coupled with an ESI source, and using the multiple reaction monitoring (MRM) mode. The ion pairs were shown in Supplementary Table 1. To ensure the retention time consistency of compounds, the liquid chromatography conditions of the pseudo-targeted analysis method were identical to those of the untargeted method. Mass spectrometry instrument parameters were as follows: the curtain gas (CUR) value was set at 40 psi; the collision gas (CAD) setting was medium; the ionspray voltage was 3500 V and the source temperature was at 320°C. The pressure for nebulization gas (GS1) and drying gas (GS2) were both set at 55 psi. The entrance potential (EP) was 10 V and collision cell exit (CXP) was 6 V.

### Metabolomics Data Processing and Statistical Analysis

All raw data from UPLC-ESI-QE-Orbitrap-MS were imported into the Progenesis QI (Version 2.4, Waters Corporation, Milford, MA, United States) software for peak extraction and peak alignment. The precise molecular mass was determined within measurement errors (<10 ppm). The variables were labeled through comparison with an in-house database of steroidal alkaloids from the *Veratrum*, and the literature (Li et al., 2007). The one-way ANOVA was used to distinguish different sources of populations. Then the false discovery rates (FDR, *q*-value) were used to control error propagation. And the peak areas of the metabolites measured with MRM were integrated by SCIEX OS software (Version 1.4, AB Sciex Corporation, Framingham, MA, United States).

Before principal component analysis (PCA) using the SIMCA-P (12.0.0.0, Umetrics AB, Umea, Sweden) software, the data of variables were scaled by unit variance (UV) and missing data were populated with half of the minimum value for each variable. According to the “80% rule” (Smilde et al., 2005; Bijlsma et al., 2006), peaks present in more than 80% of samples of either group were kept for further analysis. The missing data were still populated with half of the minimum value.

The univariate statistical analysis and data visualization processes were carried out using laboratory self-programmed software based on open-source software R and the MetaboAnalyst 5.0 website (Pang et al., 2021).

## Results and Discussion

### Molecular Assisted Identification

**FIGURE 2:**
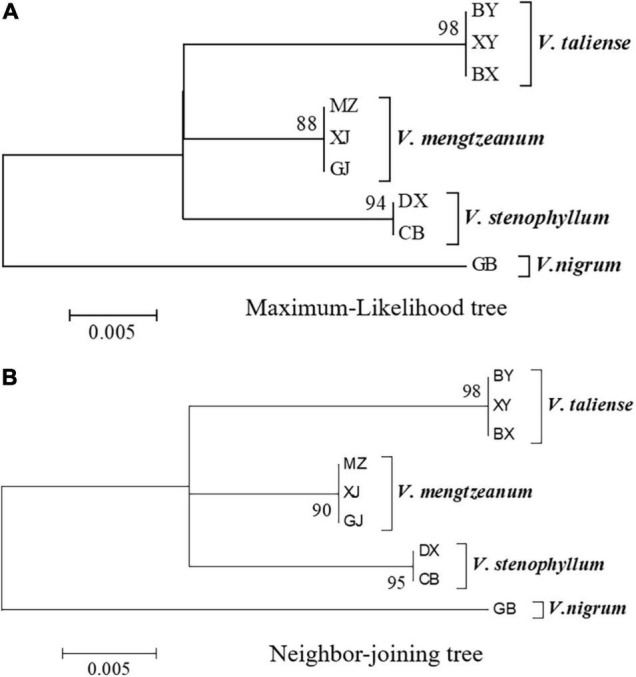
Molecular phylogenetic analysis of Pimacao origin plants. **(A)** Molecular phylogenetic analysis by the maximum-likelihood method based on the Tamura-Nei model. **(B)** Evolutionary relationships of taxa were inferred using the neighbor-Joining method. The percentage of replicate trees in which the associated taxa clustered together in the bootstrap test (1,000 replicates) is shown next to the branches.

The representative sequences of each population were registered in the Genebank with accession numbers as shown in Table 1. For molecular phylogenetic analysis, two analytical methods (neighbor-joining and maximum-likelihood) were employed. As shown in Figure 2, regardless of algorithm and model, sequences from populations GJ, MZ, and XJ formed one branch, where the evolutionary divergence between their sequences was zero (Table 2). However, there were significant differences in the morphological characteristics between GJ, MZ, and XJ (Figure 1). In 2013, Trias-Blasi and Suksathan defined two distinct subspecies, *V. mengtzeanum* subsp. *mengtzeanum* and *V. mengzeanum* subsp. *phuwae*, based on morphology and distribution. Among them, *V. mengzeanum* subsp. *phuwae* was a new subspecies (Trias-Blasi and Suksathan, 2013), which had the same morphological characteristics as the plants of XJ. The plants from XJ were then identified as *V. mengtzeanum* subsp. p*huwae*, and from GJ and MZ as *V. mengtzeanum* subsp. *mengtzeanum*. Here the new subspecies, *V. mengzeanum* subsp. *Phuwae*, was first collected in China.

**TABLE 2:** Estimates of evolutionary divergence between sequences.

	BX	BY	XY	MZ	XJ	GJ	DX	CB	GB
BX									
BY	0.000								
XY	0.000	0.000							
MZ	0.024	0.024	0.024						
XJ	0.024	0.024	0.024	0.000					
GJ	0.024	0.024	0.024	0.000	0.000				
DX	0.028	0.028	0.028	0.020	0.020	0.020			
CB	0.028	0.028	0.028	0.020	0.020	0.020	0.000		
GB	0.052	0.052	0.052	0.044	0.044	0.044	0.048	0.048	

*The number of base differences per site from between sequences is shown. The analysis involved nine nucleotide sequences. Codon positions included were 1st+2nd+3rd+Non-coding. All positions containing gaps and missing data were eliminated. Evolutionary analyses were conducted in MEGA6.*

The trees of ML and NJ (Figure 2) showed that *V. taliense*, *V. mengtzeanum*, and *V. stenophyllum* separately constituted an independent branch with high bootstrap values and were parallel to the clustered branch of *V. nigrum*. This indicated that the four species could be separated and identified by ITS2. However, ITS2 could not be used for intraspecific identification.

### Metabolite Profiling of Steroidal Alkaloids

**FIGURE 3:**
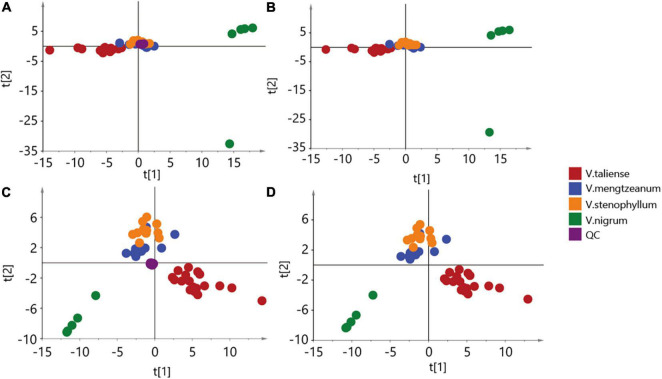
Principal component analysis (PCA) score plot. **(A,B)** The aggregation of 147 variables. **(C,D)** The aggregation of 97 variables, after clearing by the “80% rule”.

**FIGURE 4:**
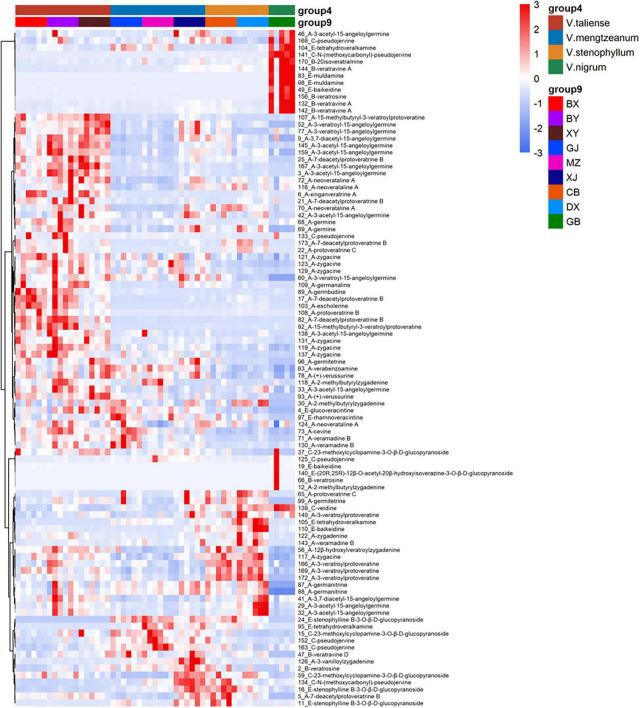
The hierarchical cluster analysis (HCA) of populations.

The results of labeled 147 variables were shown in Supplementary Table 1. As Figure 3A demonstrates, the QC samples were closely clustered, indicating good method stability. The PCA score plot without QC samples was shown in Figure 3B. After clear data according to the “80% rule” and missing data populated, 97 variables were used for further analysis. The PCA analysis results were shown in Figures 3C,D. In Figure 3D, PC1 (23.31%) and PC2 (12.66%) explained 35.97% of the variation. The *R*-value was 0.36 and the *Q*^2^-value was 0.237. This showed the overall differences of Pimacao samples from different populations and different species. Hierarchical cluster analysis (HCA) was required to find different variation features between populations. As shown in Figure 4, the pattern of clustering was similar to the pattern depicted in the Figure 2. This indicated that the labeled variables, according to steroid saponin from the *Veratrum*, were correlated with phylogenetics of the origin plants of Pimacao. Notably, *V. mengtzeanum* subsp. *mengtzeanum* and *V. mengzeanum* subsp. *phuwae* formed one branch. This was additional evidence to support the new subspecies, *V. mengzeanum* subsp. *phuwae*.

### Development and Optimization of a Multiple Reaction Monitoring Mode Method

A pseudo-targeted MS approach of these 147 variables was developed to identify potential markers. For each variable, at least two fragment ions were selected to form MRM ion pairs. The declustering potential (DP) of each analyte was 70 V and the collision energy (CE) of each analyte ion pair was optimized using QC samples. In brief, nine MRM methods with various CE values were established (CE from 10 to 50 V, increased by 5 V in turn), where 1 μL of the QC was injected. The CE value with the highest response for each analyte was selected and the analytes with more than two detected fragments were chosen. Linearity curves were then made by varying the injection volume (0.50, 0.75, 1.00, 2.00 μL) of QC samples, where each injection volume was performed in triplicate. Finally, the ion pairs and optimal CE values of 29 variables were determined by linear response of the analyte within the injection volume range. The detailed list of MRM, the linear equations of quantitative ions, and their *R*^2^ are shown in Table 3. The labeled results included 22 cevanine type (A), two veratramine type (B), and five verazine type (E). All presented good linearity, where the *R*^2^ values of 25 of the variables were higher than 0.96. Then the data was obtained with a 0.75 μL final injection volume of each sample, and a QC was also injected after every six samples running.

**TABLE 3:** Selected ion pairs used in MRM mode.

ID	RT (min)	Precursor mass	CE	Fragment mass	Quantitative ion pair	Compound ID	Linear equation	*R* ^2^
68_A	2.01	510.31	45	492.30; 151.08	510.31–492.30	68_A-germine	*y* = 718634.54x+254534.14	0.8726
71_A	2.53	478.32	50	460.30; 442.30	478.32–460.30	71_A-veramadine B	*y* = 82557429.72x+916064.26	0.9960
73_A	2.67	524.32	40	492.30; 474.29; 456.27	524.32–474.29	73_A-cevine	*y* = 650859.44x+32795.18	0.9957
118_A	3.49	578.37	40	560.36; 542.34	578.37–560.36	118_A-2-methylbutyrylzygadenine	*y* = 105540.56x+44771.49	0.9849
126_A	4.46	644.34	50	626.34; 608.33; 458.29	644.34–626.34	126_A-3-vanilloylzygadenine	*y* = 454361.45x+39240.96	0.9939
144_B	5.78	568.33	40	453.23; 273.16; 84.08	568.33–273.16	144_B-veratravine A	*y* = 105943.78x+115851.41	0.9726
149_A	6.43	690.35	50	672.34; 472.27	690.35–672.34	149_A-3-veratroylprotoveratine	*y* = 607983.94x-55232.93	0.9942
156_B	7.21	572.36	20	554.35; 457.26; 84.08	572.36–554.35	156_B-veratrosine	*y* = 7878232.93x+2277710.84	0.9959
159_A	7.45	656.34	50	596.31; 574.34	656.34–596.31	159_A-3-acetyl-15-angeloylgermine	*y* = 11429236.95x+160602.41	0.9766
169_A	9.08	654.33	40	636.32; 618.30; 165.05	654.33–636.32	169_A-3-veratroylprotoveratine	*y* = 235742.97x+69273.09	0.9824
5_A	10.93	750.41	50	732.39; 690.38; 554.31; 154.12	750.41–732.39	5_A-7-deacetylprotoveratrine B	*y* = 1088915.66x+277610.44	0.9874
9_A	11.75	698.35	50	638.33; 578.31; 478.26; 154.12	698.35–638.33	9_A-3,7-diacetyl-15-angeloylgermine	*y* = 4934297.19x+7720642.57	0.8554
16_E	13.33	578.4	50	560.39; 520.77; 98.10; 126.13; 253.20	578.40–560.39	16_E-stenophylline B-3-O-β-D-glucopyranoside	*y* = 446602409.64x+495068273.09	0.9887
29_A	14.23	634.36	45	556.32; 516.30; 474.28; 154.12	634.36–556.32	29_A-3-acetyl-15-angeloylgermine	*y* = 2756144.58x+300763.05	0.9935
32_A	14.64	634.36	45	616.35; 598.34; 556.32; 538.32	634.36–616.35	32_A-3-acetyl-15-angeloylgermine	*y* = 6968527.13x+2011666.67	0.9952
46_A	16.74	598.34	45	580.31; 562.32; 458.28	598.34–580.31	46_A-3-acetyl-15-angeloylgermine	*y* = 1080610.44x+407518.07	0.9958
49_E	17.49	474.36	40	456.35; 396.33; 98.10	474.36–98.10	49_E-baikeidine	*y* = 2600963.86x+1937309.24	0.9888
52_A	18.04	756.4	50	738.39; 638.33; 556.32; 456.28; 154.12	756.40–738.39	52_A-3-veratroyl-15-angeloylgermine	*y* = 50065863.45x+16521686.75	0.9903
60_A	18.78	778.38	50	678.33; 496.27	778.38-678.33	60_A-3-veratroyl-15-angeloylgermine	*y* = 792835.34x+314445.78	0.9616
63_A	19.16	758.41	45	740.40; 656.34; 638.33; 620.33; 165.06	758.41–740.40	63_A-verabenzoamine	*y* = 2049317.27x+1091767.07	0.9745
65_A	19.49	832.41	50	814.39; 632.34	832.41–814.39	65_A-protoveratrine C	*y* = 117765.46x+42032.53	0.9809
83_E	21.76	458.36	35	398.34; 271.21; 98.10	458.36–98.10	83_E-muldamine	*y* = 38677911.65x+9629718.88	0.9960
87_A	22.34	718.42	10	658.39; 538.31; 154.12	718.42–658.39	87_A-germanitrine	*y* = 1038377.51x+384140.56	0.8951
92_A	22.82	774.41	50	756.40; 738.39; 714.38; 596.38; 154.12	774.41–756.40	92_A-15-methylbutyryl-3-veratroylprotoveratine	*y* = 5164979.92x+818875.5	0.9900
96_A	23.15	816.42	50	798.41; 756.40; 738.39	816.42–798.41	96_A-germitetrine	*y* = 275052.21x+70923.69	0.9872
97_E	23.27	530.38	45	512.38; 494.36;	530.38–512.38	97_E-rhamnoveracintine	*y* = 5206104.42x+2518514.06	0.9884
103_A	24.52	776.42	10	758.41; 740.40; 716.39; 578.31; 154.12	776.42–758.41	103_A-escholerine	*y* = 405702.81x+88357.43	0.9139
108_A	26.72	788.42	50	670.36; 154.12	788.42–670.36	108_A-protoveratrine B	*y* = 46517.27x+1667.07	0.9668
110_E	27.06	474.36	35	456.35; 398.34; 98.10	474.36–398.34	110_E-baikeidine	*y* = 1161333.33x+259666.67	0.9956

**FIGURE 5:**
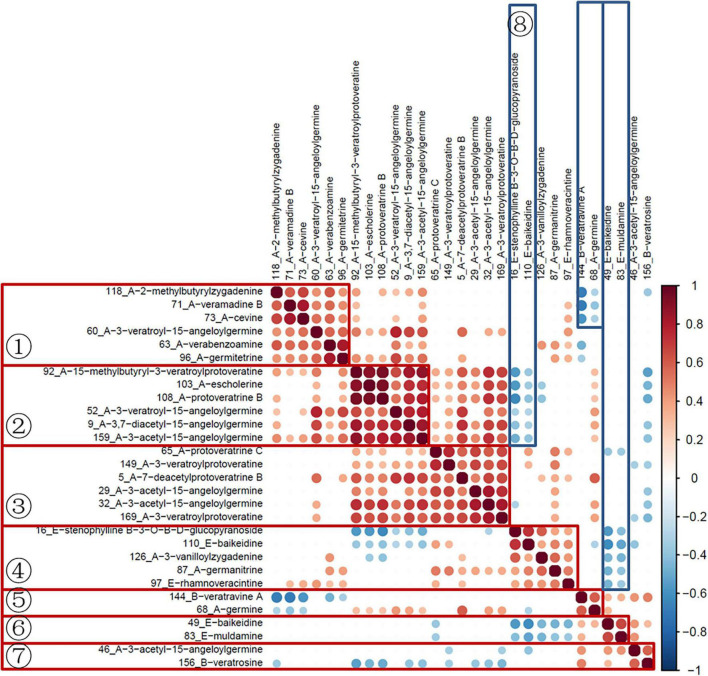
Correlation analysis of selected variables used in MRM mode.

Subsequently, the data of quantitative ion areas were integrally normalized to sample weight and shown in Supplementary Table 2, while the other ions were used for qualification. The Spearman rank test was used for correlation analysis of variables and visualized with the corrplot package (Version 0.84). As shown in Figure 5, variables in groups 1–7 had a significant positive correlation. The variables in groups 1–3 were all cevanine type (A), and groups 5 and 7 included cevanine type (A) and veratramine type (B). These two types belong to the Veratrum alkaloids, which feature a rearranged C-nor-D-homosteroidal ring structure in which the C-ring is five membered and the D-ring is six membered (Chandler and McDougal, 2014). Furthermore, there was a negative correlation between groups 8 and 2, group 5 and part of 1, as well as negative correlations between groups 6 and 4. Among these groups, 6 and 8 were the verazine type (E), which are Solanum alkaloids featuring the classic cyclopentanophenanthrene ring structure (Chandler and McDougal, 2014). More research is needed here as the biosynthesis of steroidal alkaloids in the *Veratrum* genus is not well understood (Szeliga et al., 2020).

**FIGURE 6:**
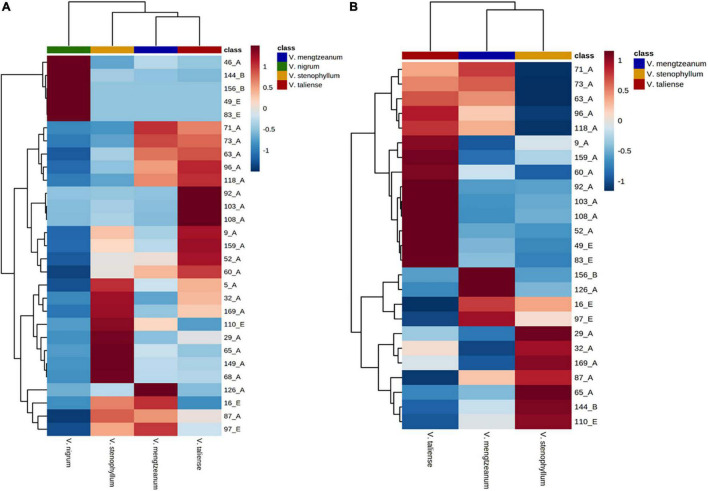
Hierarchical clustering heatmaps of selected variables. Each colored cell on the map corresponds to a concentration value. **(A)** The selected 29 variables could be used for the identification the origin plants of Pimacao. **(B)** The selected 25 variables could be used for the identification of *V. taliense*, *V. stenophyllum*, and *V. mengtzeanum*.

The hierarchical clustering heatmaps were generated for intuitive visualization, with distance measures by minkowski and clustering algorithm by average. Here, each colored cell on the map corresponds to a concentration value. As shown in Figure 6A, the selected 29 variables could be used for the identification of the original plants of Pimacao. Coupled with univariate analysis, 25 variables were selected for the identification of *V. taliense*, *V. stenophyllum*, *V. mengtzeanum* (Figure 6B).

Univariate analysis was performed to distinguish the differential variables between intraspecific populations. For *V. stenophyllum*, Student’s *t*-test or Mann-Whitney *U*-test was used according to data normality and homogeneity of variance. Whereas for *V. taliense* and *V. mengtzeanum*, a one-way ANOVA test was used to identify differences between multiple populations. A value of *p* < 0.05 was considered to be a significant difference. The results were shown in Table 4. Nine variables had a statistical difference between the populations of *V. mengtzeanum*, 14 variables between populations of *V. taliense*, and seven variables between populations of *V. stenophyllum*, respectively. It was also worth mentioning that there were 17 variables that had statistical differences between the two subspecies of *V. mengtzeanum*. However, all of these variables need further validation before employment due to the limited number of populations in this study.

**TABLE 4:** Statistical differences in selected variables between populations and subspecies.

Compound ID	Populations of *V. mengtzeanum*	*V. mengtzeanum* subsp. *mengtzeanum* and *V. mengzeanum* subsp. *phuwae*	Populations of *V. taliense*	Populations of *V. stenophyllum*
68_A-germine	*	***	*	
71_A-veramadine B		*		
73_A-cevine	*		**	
118_A-2-methylbutyrylzygadenine	*	***	*	
126_A-3-vanilloylzygadenine	*	*		
144_B-veratravine A	**	*	**	*
149_A-3-veratroylprotoveratine			*	
156_B-veratrosine	*	*		
159_A-3-acetyl-15-angeloylgermine		*	*	
169_A-3-veratroylprotoveratine		*		
5_A-7-deacetylprotoveratrine B	*	*	*	
9_A-3,7-diacetyl-15-angeloylgermine		*		
16_E-stenophylline B-3-O-β-D-glucopyranoside		*		*
29_A-3-acetyl-15-angeloylgermine			*	*
32_A-3-acetyl-15-angeloylgermine		*		*
46_A-3-acetyl-15-angeloylgermine		**		
49_E-baikeidine			*	
52_A-3-veratroyl-15-angeloylgermine	*	*	***	
60_A-3-veratroyl-15-angeloylgermine				*
63_A-verabenzoamine				
65_A-protoveratrine C			*	
83_E-muldamine			***	
87_A-germanitrine				
92_A-15-methylbutyryl-3-veratroylprotoveratine		**	***	
96_A-germitetrine				
97_E-rhamnoveracintine				
103_A-escholerine			*	**
108_A-protoveratrine B		*		
110_E-baikeidine	*	*		*
Total	9	17	14	7

*p < 0.05, **p < 0.01, ***p < 0.001.

## Conclusion

In the present study, based on phylogeny of ITS2 sequence and metabolite profiling of steroidal alkaloids, taken into account the morphological characteristics at the same time, the classification of two distinct subspecies in *V. mengzeanum* has been reinforced. And the new subspecies *V. mengzeanum* subsp. *phuwae* was collected in China for the first time. ITS2 sequence could be used for the identification of *V. taliense*, *V. mengtzeanum*, *V. stenophyllum*, and *V. nigrum* but was inefficient for intraspecific identification. Twenty-nine labeled steroidal alkaloids detected by the MRM mode could distinguish four species, while 25 labeled steroidal alkaloids could distinguish between three species that were closely related. This demonstrates that they could be used as markers for identification. Lastly, there were several variables that had a statistical difference between the two subspecies of *V. mengtzeanum* and populations of *V. taliense*, *V. mengtzeanum*, and *V. stenophyllum*.

## Data Availability Statement

The datasets presented in this study can be found in online repositories. The names of the repository/repositories and accession number(s) can be found below: https://www.ncbi.nlm.nih.gov/, MG745765, MG745766, MG745771, MG745776, MG745774, MG745768, MG745769, MG745770, and MG745767.

## Author Contributions

QL and SW: data curation, formal analysis, and writing—original draft preparation. ZY and XH: data curation and resources. QC: methodology and data curation. QW: methodology. QH: investigation. YG: formal analysis. HT: writing—reviewing and editing. HX: conceptualization, funding acquisition, project administration, and writing—reviewing. All authors agreed to be accountable for all aspects of work ensuring integrity and accuracy.

## Conflict of Interest

XH was employed by the Yunnan Baiyao Group Co., Ltd. The remaining authors declare that the research was conducted in the absence of any commercial or financial relationships that could be construed as a potential conflict of interest.

## Publisher’s Note

All claims expressed in this article are solely those of the authors and do not necessarily represent those of their affiliated organizations, or those of the publisher, the editors and the reviewers. Any product that may be evaluated in this article, or claim that may be made by its manufacturer, is not guaranteed or endorsed by the publisher.

## Supplementary Material

Supplementary Figure 1 Skeletons of steroidal alkaloids from *Veratrum* plants.

Supplementary Table 1 The results of labeled 147 variables.

Supplementary Table 2 The data of quantitative ion areas (integrally normalized to sample weight).

## Abbreviations

CAD: collision gas
ACN: acetonitrile
CUR: curtain gas
CXP: collision cell exit
dd-MS2: data-dependent MS/MS
DP: declustering potential
EP: entrance potential
ESI: electrospray ionization
FA: formic acid
HCA: hierarchical cluster analysis
Hh: Hedgehog
ITS: internal transcribed spacer
ITS2: second internal transcribed spacer
MeOH: methanol
MRM: multiple reaction monitoring
PCA: principal component analysis
QC: quality control
rDNA: ribosomal DNA
UPLC-ESI-QE-Orbitrap-MS: ultra-high-performance liquid chromatography electrospray ionization orbitrap tandem mass spectrometry
UV: unit variance.
