# Supplementary figures and images for: Identification of Veratrum Species in Pimacao Based on ITS2 Sequences and Steroidal Alkaloids by a Pseudo-Targeted Metabolomics Method
Source: Front Plant Sci. 2022 Apr 11;13:831562. doi: 10.3389/fpls.2022.831562 (PMC9037537; doi:10.3389/fpls.2022.831562)

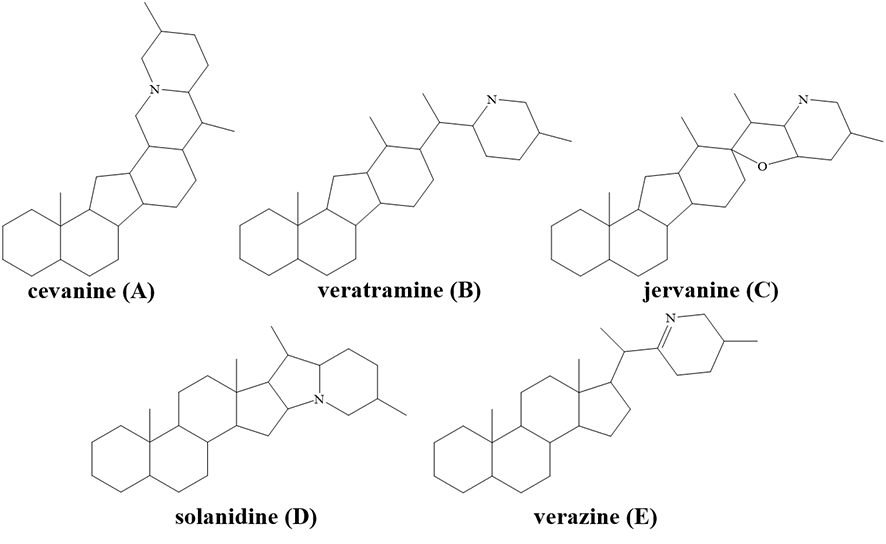

Supplement: Supplementary Figure 1 — Skeletons of steroidal alkaloids from Veratrum plants. [file Image_1.TIF]
